# Supplementary material for: Naloxegol for the Treatment of Opioid-Induced Constipation in Patients with Cancer Pain: A Pooled Analysis of Real-World Data
Source: Cancers (Basel). 2025 Mar 3;17(5):865. doi: 10.3390/cancers17050865 (PMC11898930; doi:10.3390/cancers17050865)
Supplement: Supplementary file 1 [file cancers-17-00865-s001.zip › cancers-3478537-supplementary.pdf]

cancers

MDPI

Article

Naloxegol for the Treatment of Opioid-Induced Constipation in Patients with Cancer Pain: A Pooled Analysis of Real-World Data

Jean-Marc Sabaté [1,2,*], Carmen Beato-Zambrano [3], Manuel Cobo [4], Antoine Lemaire [5], Vincenzo Montesarchio [6], Judith Serna-Montros [7], Rafik Namane [8], Santiago Martín Baccarelli [9] and Fernando Rico-Villademoros [10]

1 Gastroenterology and Gastrointestinal Oncology, Hôpital Avicenne, AP-HP, Sorbonne University, 93000 Bobigny, France
2 INSERM U987, Pathophysiology and Clinical Pharmacology of Pain, 92012 Boulogne Billancourt, France
3 Medical Oncology, Hospital Universitario Virgen Macarena, 41009 Sevilla, Spain; cbeatoz@hotmail.com
4 Medical Oncology, Instituto de Investigación Biomédica de Málaga-Plataforma BIONAND (IBIMA-BIONAND), Hospital Regional Universitario Malaga, 29010 Malaga, Spain; manuelcobodols@yahoo.es
5 Oncology & Medical Specialties Department, Valenciennes General Hospital, 59300 Valenciennes, France; lemaire-a@ch-valenciennes.fr
6 Pneumology and Oncology, A.O.R.N. dei Colli-Monaldi Hospital, 80131 Napoli, Italy; vincenzo.montesarchio@ospedalideicolli.it
7 Palliative Care/Medical Oncology Vhio, Hospital Campus Vall d'Hebron, 08035 Barcelona, Spain; judith.serna@vallhebron.cat
8 Laboratoires Grünenthal, 92800 Puteaux, France; rafik.namane@grunenthal.com
9 Data Management & Biostatistics Department, APICES, 28320 Pinto, Madrid, Spain; santiago.martin@apices.es
10 Medical Department, APICES, 28320 Pinto, Madrid, Spain; fernando.rico-villademoros@apices.es
* Correspondence: jean-marc.sabate@aphp.fr; Tel.: +33-1-48-95-55-55

Supplementary Table S1. Characteristics of the studies included in NALOPOOL.

	**KYONAL**	**MOVE**	**NACASY**
Study design	Observational, prospective<br><br>Multicenter (Spain)	Observational, prospective<br><br>Multicenter (France)	Observational, prospective<br><br>Multicenter (Europe)
Study duration	12 months	4 weeks	4 weeks
Study visIts	Week 2, Month 1, Month 3, Month 6 and Month 12	Week 4	Week 1, Week 2 and Week 4
Key inclusion criteria	(1) men/women ≥18 years of age<br><br>(2) a diagnosis of active oncological disease requiring treatment with opioids for pain control<br><br>(3) patients with symptoms of OIC at the time of screening (an average of <3 spontaneous bowel movements a week with associated symptoms of constipation in at least 25% of the bowel movements)<br><br>(4) patients with an inadequate response to laxatives for the treatment of OIC. and with indication for naloxegol<br><br>(5) patients with a Karnofsky performance status score ≥50 at study entry	1) aged ≥ 18 years with cancer pain treated with step II or III opioids for their cancer pain<br><br>2) starting naloxegol treatment for OIC (ROME IV criteria) with inadequate response to laxative(s)<br><br>An inadequate response to laxatives was defined by OIC symptoms despite the use of laxatives for at least 4 days prior to inclusion. in accordance with previous international studies	1) ≥18 years of age) with cancer pain. who had been receiving treatment with opioids for at least 4 weeks, were expected to remain on opioids for the entire duration of the study<br><br>2) had been diagnosed with OIC (defined as <3 spontaneous bowel movements. SBMs) documented per week on average in the previous 2 weeks before diagnosis. In addition. patients must have reported ≥2 of the following symptoms in at least 25% of the bowel movements (BMs) during that period: lumpy or hard stools, according to the Bristol Stool Scale (BSS); straining; sensation of incomplete BM; sensation of anorectal obstruction or blockage; a need for manual maneuvers to facilitate BMs; and, finally, loose stools rarely present without the use of laxatives.
Key exclusion criteria	Contraindication in the Summary of Product Characteristics of naloxegol and patients with cognitive impairment or uncooperative	Participating in an interventional study<br><br>Evidence of digestive obstruction	Diagnosed with colorectal cancer

OIC, opioid-induced constipation; SBM, spontaneous bowel movements

Supplementary Table S2. PAC-QoL: Physical discomfort subscale by visit.

		Physical discomfort							
**STUDY**		**N**	**Mean**	**Standard deviation**	**Minimum**	**Q1**	**Median (Q2)**	**Q3**	**Maximum**
**Kyonal**	**Baseline**	124	2,1	,9	,3	1,5	2,1	2,8	4,0
	**Week 4**	115	1,1	,8	,0	,5	1,0	1,8	4,0
**Move**	**Baseline**	110	2,0	1,0	,0	1,3	2,0	2,8	4,0
	**Week 4**	77	1,3	,9	,0	,8	1,3	1,8	4,0
**Nacasy**	**Baseline**	139	2,1	,9	,0	1,5	2,3	2,8	4,0
	**Week 4**	114	1,2	,9	,0	,5	1,0	1,8	4,0
**Total**	**Baseline**	373	2,1	,9	,0	1,5	2,0	2,8	4,0
	**Week 4**	306	1,2	,9	,0	,5	1,0	1,8	4,0

Supplementary Table S3. PAC-QoL: Psychosocial discomfort subscale by visit.

		Psychosocial discomfort							
**STUDY**		**N**	**Mean**	**Standard deviation**	**Minimum**	**Q1**	**Median (Q2)**	**Q3**	**Maximum**
**Kyonal**	**Baseline**	124	1,5	1,0	,0	,8	1,6	2,3	3,8
	**Week 4**	115	,8	,8	,0	,1	,6	1,3	3,5
**Move**	**Baseline**	110	1,5	,9	,0	,8	1,4	2,0	3,5
	**Week 4**	77	,9	,8	,0	,3	,8	1,3	4,0
**Nacasy**	**Baseline**	139	1,5	1,0	,0	,8	1,6	2,3	3,6
	**Week 4**	114	,7	,7	,0	,1	,5	1,1	3,6
**Total**	**Baseline**	373	1,5	1,0	,0	,8	1,5	2,1	3,8
	**Week 4**	306	,8	,8	,0	,3	,6	1,3	4,0

Supplementary Table S4. PAC-QoL: Worries and concerns subscale by visit.

		Worries and concerns							
STUDY		N	Mean	Standard deviation	Minimum	Q1	Median (Q2)	Q3	Maximum
**Kyonal**	**Baseline**	124	2,2	,8	,5	1,5	2,3	2,8	4,0
	**Week 4**	115	1,4	,9	,0	,6	1,2	1,9	4,0
**Move**	**Baseline**	110	2,3	,8	,5	1,7	2,3	2,9	4,0
	**Week 4**	77	1,7	,9	,2	1,1	1,6	2,2	3,9
**Nacasy**	**Baseline**	139	2,0	,8	,2	1,4	2,1	2,7	4,0
	**Week 4**	114	1,2	,8	,0	,5	1,2	1,8	3,0
**Total**	**Baseline**	373	2,2	,8	,2	1,5	2,3	2,8	4,0
	**Week 4**	306	1,4	,8	,0	,7	1,4	2,0	4,0

Supplementary Table S5. PAC-QoL: Satisfaction subscale by visit.

		Satisfaction							
STUDY		N	Mean	Standard deviation	Minimum	Q1	Median (Q2)	Q3	Maximum
**Kyonal**	**Baseline**	124	2,6	,4	1,4	2,2	2,6	3,0	3,2
	**Week 4**	115	1,8	,6	,6	1,4	1,8	2,2	3,2
**Move**	**Baseline**	109	2,7	,5	1,2	2,6	2,8	3,0	4,0
	**Week 4**	76	2,1	,6	,8	1,7	2,0	2,6	3,2
**Nacasy**	**Baseline**	139	2,5	,5	1,2	2,2	2,6	3,0	3,2
	**Week 4**	114	2,0	,6	,8	1,6	2,0	2,4	3,2
**Total**	**Baseline**	372	2,6	,5	1,2	2,2	2,6	3,0	4,0
	**Week 4**	305	1,9	,6	,6	1,4	2,0	2,4	3,2

Supplementary Table S6. PAC-QoL: Global score by visit.

STUDY		N	Mean	Standard deviation	Minimum	Q1	Median (Q2)	Q3	Maximum
		Global score							
Kyonal	Baseline	124	2,1	,7	,7	1,4	2,1	2,6	3,7
	Week 4	115	1,2	,7	,1	,7	1,2	1,7	3,7
Move	Baseline	110	2,1	,6	,8	1,6	2,1	2,6	3,4
	Week 4	77	1,5	,7	,3	,9	1,5	1,8	3,5
Nacasy	Baseline	139	2,0	,7	,5	1,5	2,0	2,5	3,4
	Week 4	114	1,2	,7	,1	,8	1,0	1,7	3,3
Total	Baseline	373	2,0	,7	,5	1,5	2,1	2,6	3,7
	Week 4	306	1,3	,7	,1	,8	1,2	1,8	3,7

Supplementary Table S7. PAC-SYM: Abdominal symptom subscale by visit.

			PAC-SYM: Abdominal symptom subscale							
			N	Mean	Standard deviation	Minimum	Q1	Median (Q2)	Q3	Maximum
STUDY ID	Kyonal	Baseline	124	1,5	,9	,0	1,0	1,3	2,0	4,0
		Week 4	117	,8	,7	,0	,3	,8	1,3	3,8
	Move	Baseline	110	1,8	1,0	,0	1,3	1,9	2,5	4,0
		Week 4	77	1,1	,9	,0	,5	1,0	1,8	3,5
	Total	Baseline	234	1,6	,9	,0	1,0	1,5	2,3	4,0
		Week 4	194	,9	,8	,0	,3	,8	1,3	3,8

Supplementary Table S8. PAC-SYM: Rectal symptom subscale by visit.

			PAC-SYM: Rectal symptom subscale							
			N	Mean	Standard deviation	Minimum	Q1	Median (Q2)	Q3	Maximum
STUDY ID	Kyonal	Baseline	124	1,4	1,0	,0	,7	1,2	2,0	4,0
		Week 4	117	,5	,7	,0	,0	,3	,7	3,0
	Move	Baseline	110	1,5	1,2	,0	,7	1,3	2,0	4,0
		Week 4	76	1,0	1,0	,0	,2	,7	1,7	4,0
	Total	Baseline	234	1,4	1,1	,0	,7	1,3	2,0	4,0
		Week 4	193	,7	,9	,0	,0	,3	1,0	4,0

Supplementary Table S9. PAC-SYM: Stool symptom subscale by visit.

			PAC-SYM: Stool symptom subscale							
			N	Mean	Standard deviation	Minimum	Q1	Median (Q2)	Q3	Maximum
STUDY ID	Kyonal	Baseline	124	2,2	,9	,0	1,6	2,2	3,0	4,0
		Week 4	117	1,0	,9	,0	,2	,8	1,6	3,6
	Move	Baseline	110	2,5	,8	,0	2,0	2,6	3,0	4,0
		Week 4	76	1,5	,9	,0	,8	1,4	2,1	4,0
	Total	Baseline	234	2,4	,9	,0	1,8	2,4	3,0	4,0
		Week 4	193	1,2	1,0	,0	,4	1,0	1,8	4,0

Supplementary Table S10. PAC-SYM: Total score by visit.

			PAC-SYM: Total score							
			N	Mean	Standard deviation	Minimum	Q1	Median (Q2)	Q3	Maximum
STUDY ID	Kyonal	Baseline	124	1,6	,7	,2	1,1	1,6	2,0	3,7
		Week 4	117	,7	,7	,0	,2	,6	1,2	2,6
	Move	Baseline	110	1,8	,7	,1	1,3	1,8	2,3	3,6
		Week 4	77	1,1	,7	,0	,6	1,0	1,6	3,3
	Total	Baseline	234	1,7	,7	,1	1,3	1,7	2,2	3,7
		Week 4	194	,9	,7	,0	,3	,8	1,3	3,3

Supplementary Table S11. BFI 1: Ease of defecation by visit.

			BFI.1: Ease of defecation							
			N	Mean	Standard deviation	Minimum	Q1	Median (Q2)	Q3	Maximum
STUDY	Move	Baseline	117	71,5	24,0	,0	50,0	80,0	90,0	100,0
		Week 4	83	44,9	28,1	,0	20,0	50,0	70,0	100,0
	Nacasy	Baseline	142	68,9	23,5	,0	50,0	70,0	85,0	100,0
		Week 4	116	40,3	27,2	,0	20,0	40,0	60,0	90,0
	Total	Baseline	259	70,1	23,7	,0	50,0	75,0	90,0	100,0
		Week 4	199	42,2	27,6	,0	20,0	45,0	65,0	100,0

Supplementary Table S12. BFI 2: Feeling incomplete evacuation by visit.

			BF.2: Feeling incomplete evacuation							
			N	Mean	Standard deviation	Minimum	Q1	Median (Q2)	Q3	Maximum
STUDY	Move	Baseline	118	65,8	29,2	,0	50,0	70,0	90,0	100,0
		Week 4	83	37,6	29,6	,0	10,0	30,0	50,0	100,0
	Nacasy	Baseline	142	66,2	28,6	,0	50,0	70,0	90,0	100,0
		Week 4	117	43,2	28,4	,0	20,0	43,0	60,0	100,0
	Total	Baseline	260	66,0	28,8	,0	50,0	70,0	90,0	100,0
		Week 4	200	40,9	29,0	,0	20,0	40,0	60,0	100,0

Supplementary Table S13. BFI 3: Self judgement of constipation by visit.

			BFI.3: Judgement of constipation							
			N	Mean	Standard deviation	Minimum	Q1	Median (Q2)	Q3	Maximum
STUDY	Move	Baseline	118	76,4	24,1	,0	60,0	80,0	100,0	100,0
		Week 4	83	37,1	29,4	,0	10,0	30,0	50,0	100,0
	Nacasy	Baseline	142	71,0	25,1	,0	60,0	80,0	90,0	100,0
		Week 4	117	39,5	29,7	,0	10,0	40,0	65,0	100,0
	Total	Baseline	260	73,5	24,7	,0	60,0	80,0	92,5	100,0
		Week 4	200	38,5	29,5	,0	10,0	40,0	60,0	100,0

Supplementary Table S14. BFI total score by visit.

			BFI: Total score							
			N	Mean	Standard deviation	Minimum	Q1	Median (Q2)	Q3	Maximum
STUDY	Move	Baseline	118	71,2	19,6	10,0	56,7	73,3	86,7	100,0
		Week 4	83	39,9	26,9	,0	17,3	40,0	60,0	100,0
	Nacasy	Baseline	142	68,7	20,4	6,7	56,7	70,0	83,3	100,0
		Week 4	117	41,0	26,5	,0	20,0	41,7	60,0	96,7
	Total	Baseline	260	69,8	20,0	6,7	56,7	70,0	85,8	100,0
		Week 4	200	40,6	26,6	,0	20,0	40,0	60,0	100,0
